# Effects of high mobility group protein box 1 and toll like receptor 4 pathway on warts caused by human papillomavirus

**DOI:** 10.3892/mmr.2014.2477

**Published:** 2014-08-11

**Authors:** HUI WENG, HONGBO LIU, YUNHUA DENG, YUYAN XIE, GUANXIN SHEN

**Affiliations:** 1 Department of Dermatology, The Fifth Affiliated Hospital of Sun Yat-Sen University, Zhuhai, Guangdong 519000, P.R. China; 2 Department of Immunology, Tongji Medical College, Huazhong University of Science and Technology, Wuhan, Hubei 430030, P.R. China; 3 Department of Dermatology, Tongji Hospital, Tongji Medical College, Huazhong University of Science and Technology, Wuhan, Hubei 430030, P.R. China; 4 Department of Pathology, The Fifth Affiliated Hospital of Sun Yat-Sen University, Zhuhai, Guangdong 519000, P.R. China

**Keywords:** high mobility group protein box 1, toll like receptor 4, nuclear factor-κB, inflammation, verruca vulgaris, condyloma acuminatum

## Abstract

Accumulative evidence has demonstrated that inflammation has an important role in human papillomavirus (HPV) oncogenicity. However, the effects of high mobility group protein box 1 (HMGB1)-toll like receptor 4 (TLR4) signaling pathway associated inflammation on epidermal warts caused by HPV remain unclear. The present study investigated the HMGB1, TLR4 and nuclear factor-κB p65 expression in condyloma acuminatum (CA) and verruca vulgaris (VV). Immunohistochemistry and western blot analysis revealed that p65 expression in epithelial nuclei in VV and CA was significantly higher than in normal skin (NS) (P<0.01), and p65 in CA was higher than in VV but this difference was not significant. The level of extracellular HMGB1 increased significantly and progressively from NS to VV to CA (P<0.05). The level of TLR4 on the surface of epithelial membranes in the CA samples was significantly higher than in NS (P<0.01), and TLR4 in VV samples was significantly lower than in NS (P<0.01). There was a positive correlation between p65 expression in the epithelial nuclei and HMGB1 in the epithelial intercellular spaces (r=0.5199, P<0.01). These findings indicate that inflammation is intensified in warts caused by HPV. HMGB1-TLR4 pathway-associated inflammation may therefore have a pivotal role in CA. HMGB1, rather than TLR4, may be a vital mediator of inflammation in VV. Therapies targeting HMGB1 may be a potential strategy for the treatment of HPV-associated warts.

## Introduction

Human papillomaviruses (HPVs) pertain to the *Papillomaviridae* family, a highly diverse group of viruses including >100 different types, and infect the epithelial cells of several vertebrate species (1). Condyloma acuminatum (CA) and verruca vulgaris (VV) are possibly the most common epidermal infection caused by HPVs. CA, defined as genital warts, in ~90% of cases is caused by the low risk HPV types 6 or 11, while the high risk HPV 16 or 18 mainly account for the rest. CA is regarded as a benign tumor and may be contagious during sexual activity. VV, which is caused mainly by HPV type 2, causes recognizable warts that are more frequently observed in children, occurring on the fingers and hands. VV is also benign and does not involve internal organs. The genesis and development mechanism of the warts caused by HPV infection is complex, and avoiding the host immune response to HPVs has been considered a key factor in triggering their development (2). Furthermore, local inflammatory responses may promote neoplasia progression through a number of different mechanisms (3,4).

HMGB1 is a fundamental nuclear DNA binding protein (5) that is secreted into the extracellular fluid to act as a proinflammatory cytokine. It binds to TLR4 to activate nuclear factor-κB (NF-κB) (6,7), which is involved in tumor progression (8). It has been recognized that HMGB1 has important roles in inflammation and cancer (9,10). Inflammation has been found to be intensified along with the increase in epidermal tumor malignancy, and the TLR4 signaling pathway has been considered to be a significant mediator of inflammation in a number of malignant tumors (11).

Until now, the role of HMGB1, TLR4 and p65 in warts caused by HPVs has not been elucidated. In the present study, their involvement in warts caused by HPV was investigated, primarily by examining the relation between HMGB1-TLR4 pathway-associated inflammation and the development of CA and VV.

## Materials and methods

### Samples

The specimens were obtained from 30 cases of CA and 19 cases of VV, which were diagnosed based on clinically apparent warts and histopathological features. The surrounding tissue of the warts in 20 participants was used as normal contrasts. The participants with immunological deficiency diseases were excluded. A therapeutic washout period of three weeks for the warts was implemented prior to specimen collection.

The study was performed in accordance with the Declaration of Helsinki of 1964 and the subsequent amendments. The Ethics Board of Tongji Medical College (Wuhan, China) and the Ethics Board of Sun Yat-Sen Medical College (Zhuhai, China) approved the experimental procedures. All of the subjects provided written informed consent prior to participating in the study.

### Antibodies and reagents

The antibodies and reagents used in the study included: Anti-HMGB1 (2600-1; Epitomics, Burlingame, CA, USA), anti-TLR4 (ab22048; Abcam, Cambridge, UK), anti-NF-κB p65 (SC-7151; Santa Cruz Biotechnology, Inc., Santa Cruz, CA, USA) and REALTMEn Vision Detection kit (Dako, Carpinteria, CA, USA).

### Immunohistochemistry

Immunohistochemistry EnVision was used to detect the expression of HMGB1, TLR4 and NF-κB p65 in wart and normal skin (NS) specimens. The 69 tissue specimens were routinely fixed in formalin and embedded in paraffin. Sections of 4 μm thickness were cut from paraffin-embedded tissue blocks and mounted on silanized slides. Following dewaxing and rehydration, the sections were antigen retrieved with ethylenediamine tetraacetic acid or citric acid, incubated with 3% H_2_O_2_ for 10 min and blocked with 5% bovine serum albumin for 20 min. The specimens were then incubated with the primary antibodies (anti-HMGB1 1:800, anti-TLR4 1:200 and anti-NF-κB p65 1:200) for 24 h at 4°C. Then, the secondary antibodies were added (ChemMate TMEnVision +/ HRP-goat anti-mouse/rabbit secondary antibody, K5007; Dako, Glostrup, Denmark) and the specimens were incubated for 45 min, followed by the addition of 50–100 μl diaminobenzidine. Dehydration, transparence and mounting of specimens for examination were conducted using routine procedures. The specimens were photographed with a Nikon Eclipse Ti-SR microscope equipped with a Nikon DS-U3 digital camera (Nikon Incorporation, Tokyo, Japan). The negative controls were obtained by omitting the primary antibodies.

Two blinded pathologists conducted semi-quantitative immunohistochemical grading of the nuclei, cytoplasm, cell membranes, whole cells or intercellular spaces. The average scores were used for analysis. The scoring system was as follows: 0, no staining; 1, light brown yellow staining; 2, brown staining; and 3, dark brown staining. Ten fields of view were counted on each slide at a magnification of ×400. The average positive expression on each slide was scored as: 1, <25%; 2, 25–49%; 3, 50–74%; and 4, ≥75%. The product of the percentage of positive expression and the degree of staining scores for each slide provided a final score, which was used to indicate the following: 0–1 point, negative (−); 2–3 points, weakly positive (+); 4–6, moderately positive (++); and >6, strongly positive (+++).

### Western blot analysis

Western immunoblot analysis was used to detect the expression of NF-κB p65 in nuclei isolated from HPV-infected and normal tissues. Dermal and subcutaneous tissues were removed from the specimens and the epidermis was sliced into small sections to prepare the epithelial nuclear proteins using a cytoplasmic/nuclear protein extraction kit (Beyotime Co., Shanghai, China). Equal quantities of cytoplasmic and nuclear extracts were subjected to 10% sodium dodecyl sulfate-polyacrylamide gel electrophoresis and transferred to nitrocellulose membranes. The membranes were blocked overnight at 4°C in phosphate-buffered saline and 0.1% Tween-20 buffer containing 5% non-fat dried milk proteins. The membranes were then blotted for 2 h at room temperature with the primary anti-NF-κB p65 antibody diluted at 1:500. The membrane-bound protein-antibody complexes were labeled with horseradish peroxidase-conjugated anti-IgG diluted at 1:3,000. Histone H3 was used as an equal protein loading control. An enhanced chemiluminescence system (Pierce Biotechnology, Inc., Rockford, IL, USA) was used for detection.

### Statistical analysis

Data are expressed as the mean ± standard error of the mean (SEM). The between-group differences were analyzed by one-way analysis of variance (ANOVA) followed by the Bonferroni method for normally distributed datasets. The Kruskal-Wallis test followed by the Nemenyi multiple comparisons test was used for skewed datasets. Spearman’s correlation test was used for the correlation analysis. P<0.05 and P<0.01 were considered to indicate a statistically significant difference. Statistical analysis was performed using R software, v3.0 (GNU Project, Boston, MA, USA).

## Results

### Expression of HMGB1 in warts caused by HPV

**Figure 1:**
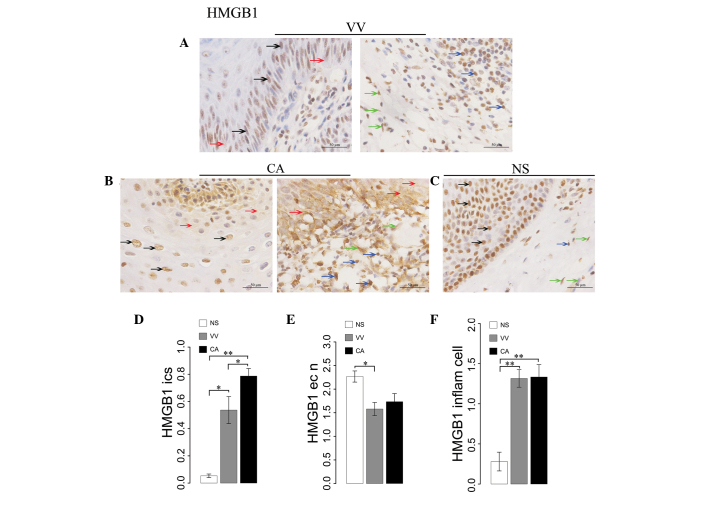
HMGB1 expression in NS and wart samples determined by immunohistochemistry EnVision (magnification, ×400). (A to C) HMGB1 positive expression is located in the nuclei, cytoplasm, cells and/or intercellular spaces following stimulation of cell necrosis or inflammation. The red arrows represent HMGB1 expression in the epithelial intercellular spaces, the black arrows represent HMGB1 expression in the epithelial cell nuclei, the blue arrows represent HMGB1 expression in inflammatory cells, and the green arrows represent HMGB1 expression in the vascular endothelial cells. (D) HMGB1 expression in the epithelial intercellular spaces. (E) HMGB1 expression in the epithelial cell nuclei. (F) HMGB1 expression in the inflammatory cells. The error bars represent the standard error of the mean. ^*^P<0.05 and ^**^P<0.01. HMGB1, high mobility group protein box 1; CA, condyloma acuminatum; VV, verruca vulgaris; NS, normal skin.

In VV, there was diffuse weakly to moderately positive expression of HMGB1 in the epithelial nuclei and weak expression in the cytoplasm. A weak expression level of extracellular HMGB1 was also found in the intercellular spaces of the epithelium. As for the associated inflammatory and vascular endothelial cells, moderately positive HMGB1 expression was observed in the nuclei and cytoplasm from VV samples (Fig. 1A).

In CA, there was diffuse moderately positive expression of HMGB1 in the epithelial nuclei but minimal expression of HMGB1 in the cytoplasm and weakly positive expression of extracellular HMGB1 in the epithelial intercellular spaces. There was moderately positive HMGB1 expression in the nuclei and cytoplasm of the associated inflammatory cells and vascular endothelial cells (Fig. 1B).

In the NS, HMGB1 exhibited moderately to strongly positive diffuse expression in the squamous epithelial nuclei and occasionally focal expression in the squamous epithelial cytoplasm. Weak extracellular HMGB1 expression in the intercellular spaces of the normal squamous epithelium was observed. There were a few inflammatory cells in the NS, which exhibited a weak HMGB1 expression in the nuclei or cytoplasm, and there was moderately to strongly positive HMGB1 expression in the nuclei and cytoplasm of vascular endothelial cells (Fig. 1C).

ANOVA demonstrated that the expression of HMGB1 in epithelial intercellular spaces of VV samples was significantly higher than in the NS (P=0.0308), and that the expression of HMGB1 in CA was significantly higher than in the NS and VV samples (P=0.0000 and P=0.0399, respectively; Fig. 1D). ANOVA also revealed that the HMGB1 expression level in the epithelial nuclei of the cells in the VV sample was significantly lower than in the NS (P=0.0194), and there was no statistically significant difference between HMGB1 in the epithelial nuclei of VV and CA samples (Fig. 1E). In addition, the expression of HMGB1 in the associated inflammatory cells of VV and CA was significantly higher than in the NS (P=0.0001 and P=0.0000, respectively). No significant difference was identified between the HMGB1 expression in the inflammatory cells in VV and CA samples (Fig. 1F).

### Expression of TLR4 in wart samples

**Figure 2:**
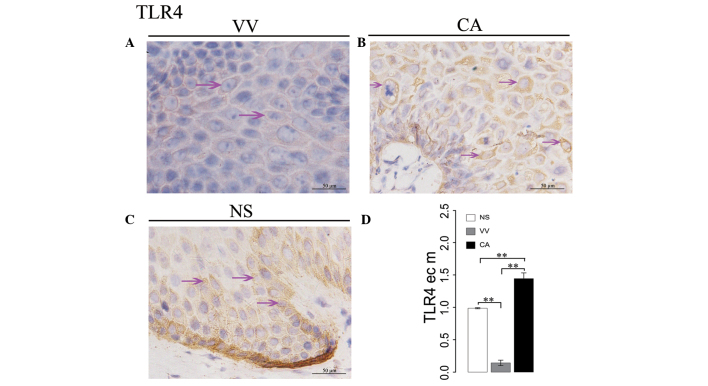
TLR4 expression in NS and wart samples by immunohistochemistry EnVision (magnification, ×400). (A to C) Purple arrows represent TLR4 expression on the epithelium membranes. (D) ^**^P<0.01 of TLR4 on epithelial cell membranes in the different tissues. The error bars represent the standard error of the mean. TLR4, toll like receptor 4; CA, condyloma acuminatum; VV, verruca vulgaris; NS, normal skin.

In VV samples, only weak TLR4 expression was observed on the surface of epithelial cell membranes (Fig. 2A). In CA samples, there was a weak to moderately positive TLR4 expression on the surface of epithelial membranes (Fig. 2B). In the NS, weakly positive TLR4 expression was observed on the surface of the epithelial cell membranes (Fig. 2C). ANOVA demonstrated that the expression of TLR4 on epithelial cell membranes in CA samples was significantly higher than in the NS (P=0.0010), and TLR4 on epithelial membranes in VV samples was significantly lower than in the NS (P=0.0001; Fig. 2D).

### Expression of NF-κB p65 in warts

**Figure 3:**
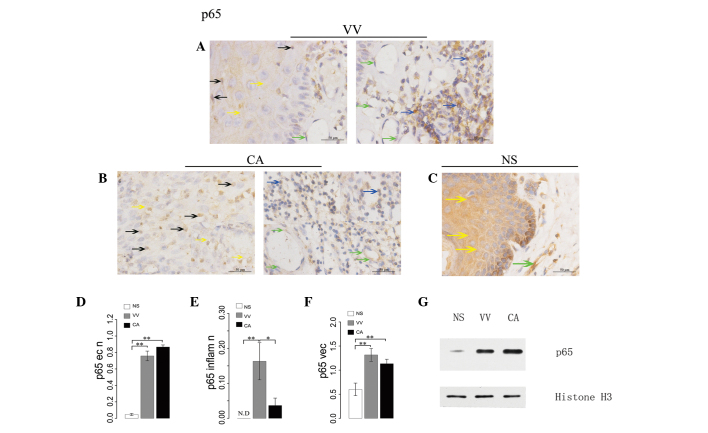
p65 expression in NS and wart samples by immunohistochemistry EnVision (magnification, ×400). (A-C) p65 positive expression is located in the cells, cytoplasm and/or nuclei following activation. The black arrows represent p65 expression in the epithelial nuclei, the yellow arrows represent p65 expression in the epithelial cytoplasm, the blue arrows represent p65 expression in inflammatory cells and the green arrows represent p65 expression in vascular endothelial cells. (D) p65 expression in the epithelial nuclei. (E) p65 expression in inflammatory cell nuclei. (F) p65 expression in the vascular endothelial cells. The error bars represent the standard error of the mean. ^*^P<0.05 and ^**^P<0.01. (G) Western blot detection of p65 expression in the epithelial nuclei, which increased from NS to VV to CA. Histone H3 was adopted as a loading control. CA, condyloma acuminatum; VV, verruca vulgaris; NS, normal skin.

In VV samples, p65 exhibited weakly positive expression in the epithelial nuclei and diffuse moderately positive expression in the cytoplasm. In the associated inflammatory cells, relatively high p65 expression was observed in the nuclei and a weakly to moderately positive p65 expression in the cytoplasm was noted. There was weakly to moderately positive p65 expression in the associated vascular endothelial cells (Fig. 3A).

CA samples exhibited weakly positive p65 expression in the nuclei and diffuse positive expression in the cytoplasm. In the associated inflammatory cells, minimal p65 expression in the nuclei and sporadic weakly positive expression in the cytoplasm was identified. In addition, there was weakly positive p65 expression in the associated vascular endothelial cells. (Fig. 3B).

In the NS, there was only minimal p65 expression in the epithelial nuclei and focal positive expression in the epithelial cytoplasm. Almost no p65 expression was observed in the nuclei of associated inflammatory cells, and a degree of focal p65 expression was observed in the cytoplasm of the associated vascular endothelial cells (Fig. 3C).

ANOVA demonstrated that the expression of p65 in the epithelial nuclei of cells from VV and CA samples was significantly higher than that in the NS (P=0.0001 and P=0.0000, respectively), and that the p65 level in CA was higher than in VV samples, however this difference was not significant (Fig. 3D). By contrast, the expression of p65 in the inflammatory cell nuclei of VV samples was significantly higher than in the NS (P=0.0049) and than in the CA samples (P=0.0412). There was no significant difference between p65 expression of inflammatory cell nuclei in CA and NS samples (Fig. 3E). ANOVA also demonstrated that the expression of p65 in vascular endothelial cells of NS was significantly lower than in VV (P=0.0007) and CA (P=0.0069) samples, and there was no significant difference in p65 expression in the vascular endothelial cells between VV and CA (Fig. 3F).

Western immunoblot analysis identified different levels of p65 expression in the epithelial nuclei of the NS and the wart specimens. There was only a weak p65 expression in the epithelial nuclei of the NS, whereas the epithelial nuclei of CA and VV demonstrated strong expression. In addition, p65 epithelial nuclear expression in CA was higher than that observed in VV (Fig. 3G).

### Correlation analysis

**Figure 4:**
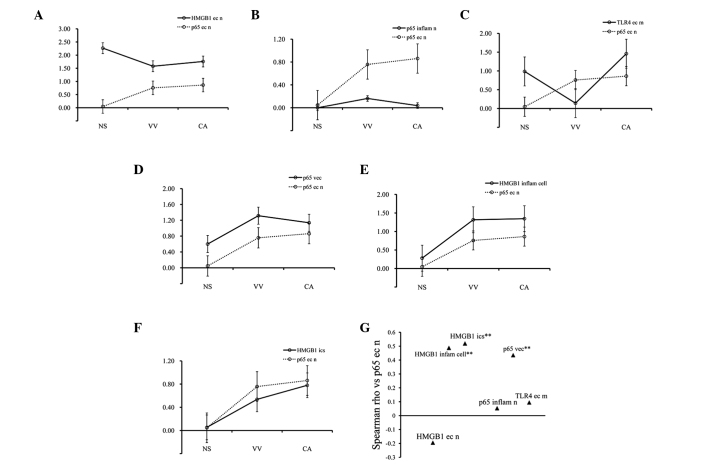
Correlation analysis of immunohistochemistry. (A–F) The line-charts with the standard error of the mean represent p65 expression in the epithelial cell nuclei and other factors by Spearman’s correlation analysis. (G) The Spearman’s correlation coefficients as compared with p65 in epithelial cell nuclei. (A and G) p65 in epithelial cell nuclei vs. HMGB1 in the epithelial nuclei (r=−0.1947, P>0.05). (B and G) p65 in epithelial cell nuclei vs. p65 in the inflammatory nuclei (r=0.0536, P>0.05). (C and G) p65 in epithelial cell nuclei vs. TLR4 on the epithelial cell membranes (r=0.0949, P>0.05). (D and G) p65 in epithelial cell nuclei vs. p65 in the vascular endothelial cells (r=0.4355, ^**^P<0.01). (E and G) p65 in epithelial cell nuclei vs. HMGB1 in the inflammatory cells (r=0.4879, ^**^P<0.01). (F and G) p65 in epithelial cell nuclei vs. HMGB1 in the epithelial intercellular spaces (r=0.5199, ^**^P<0.01). HMGB1, high mobility group protein box 1; TLR4, toll like receptor 4; CA, condyloma acuminatum; VV, verruca vulgaris; NS, normal skin.

Spearman’s correlation analysis demonstrated that the expression of p65 in the epithelial nuclei of the NS and the CA and VV samples was negatively correlated with the HMGB1 level in the epithelial cell nuclei (r=−0.1947, P>0.05; Fig. 4A and G), was positively correlated with p65 in the inflammatory cell nuclei (r=0.0536, P>0.05; Fig. 4B and 4G), was positively correlated with TLR4 on the surface of the epithelial cell membranes (r=0.0949, P>0.05; Fig. 4C and G), with p65 in the vascular endothelial cells (r=0.4355, P=0.0002; Fig. 4D and G), with HMGB1 in the inflammatory cells (r=0.4879, P=0.0000; Fig. 4E and G) and with HMGB1 in the epithelial intercellular spaces (r=0.5199, P=0.0000; Fig. 4F and G).

## Discussion

CA and VV are the most common epidermal infection caused by HPVs. The majority of HPV infections are eliminated by an effective immune response (12,13). Failure to eradicate HPVs promotes wart development and is mainly associated with the ability of HPV to avoid the immune defense system of the body (2). This process also involves a series of inflammatory responses, which have been identified in recent years. Pro-inflammatory cytokines, including several interleukins and tumor necrosis factor, are important mediators of the development of skin and mucosal inflammation (14,15). There is evidence that epidermal cells are able to produce cytokines in response to diverse stimuli, including viral infection (14). However, data detailing the role of the HMGB1-TLR4 pathway-associated inflammation in the HPV-related epidermal warts are lacking. The present study focused on the HMGB1 expression in the epithelial intercellular spaces, TLR4 expression on the epithelial cell membrane surface and p65 expression in the epithelial nuclei in VV and CA.

HMGB1 is an essential component of mammalian chromatin in the nuclei (16). It is able to reach the intercellular spaces either through passive release from necrotic cells (17), or active secretion by macrophages and monocytes (18). Extracellular HMGB1 is a novel pro-inflammatory cytokine, which acts by binding to TLR4, TLR2 or receptors for advanced glycation end-products (19,20). The immunohistochemistry results of the present study revealed that HMGB1 expression was present in the epithelial intercellular spaces in CA and VV samples as well as in the NS, and its expression level increased significantly and progressively from the NS to VV to CA (P<0.05). These findings imply that intracellular HMGB1 is released outside of the HPV-infected cells to act as an extracellular mediator of local inflammation. The difference in extracellular HMGB1 expression between CA and VV, may be due to the fact that the two tumors are caused by variant HPV types. Expectedly, HMGB1 epithelial nuclear expression in the NS was significantly higher than in VV (P<0.05), and there was no significant difference identified in the HMGB1 epithelial nuclear expression between VV and CA. This result is in accordance with our recent study investigating the HMGB1 expression in a number of malignant epidermal tumors (11). This result also suggests that HMGB1 expression in the epithelial nuclei may have a function in the stabilization of DNA and chromosomes rather than acting as a factor that leads to wart development. In addition, HMGB1 expression in the associated inflammatory cells in VV and CA samples was higher than in the NS (P<0.01), and the correlation analysis demonstrated that the level of HMGB1 in inflammatory cells was positively correlated with the level of p65 in the epithelial nuclei (r=0.4879, P<0.01). These results suggest that HMGB1 may have a key role in inflammation in the HPV-associated warts.

Extracellular HMGB-1 has been recognized to be involved in the activation of NF-κB through its receptor TLR4 (7). The interference with the expression of TLRs may effect the activation of antigen presenting cells and phagocytes to enhance innate and adaptive responses against pathogens, and therefore induce tumorigenesis and development (21,22). From the present results that demonstrated the signifcantly lower expression of TLR4 on the surface of epithelial membranes in VV compared to normal skin (P<0.01), it is deduced that TLR4 signaling may not be involved in inducing inflammation in VV. Instead, the impairment of TLR4 may be a factor for the development of VV. The present study also demonstrated that the TLR4 expression on the surface of the epithelial membrane in CA samples was significantly higher than that of the NS (P<0.01), and that the extracellular HMGB1 expression in CA was significantly higher than in the NS (P<0.01). These findings indicate that extracellular HMGB1 may act as a pro-inflammatory factor to interact with TLR4, and that the HMGB1-TLR4 signaling pathway-associated inflammation may have a significant role in CA.

The NF-κB family mainly includes RELA (p65), NF-κB1 (p50; p105), NF-κB2 (p52; p100), c-REL and RELB (23,24). NF-κB is sequestered in the cytoplasm inactively and bound by the inhibitor proteins (24). Activated by various stimuli, including different pro-inflammatory cytokines, NF-κB is transported to the nuclei, where it binds with sequences of various genes to induce the expression of inflammatory factors, promote cell proliferation and prevent apoptosis, and may therefore have a role in tumorigenesis and tumor development (25,26). A previous study demonstrated that NF-κB expression and activation are abnormal in human epidermal tumors of seborrheic keratosis, basal cell carcinoma and squamous cell carcinoma (11). The present study revealed that there was weak positive expression of p65 in the epithelial nuclei of VV and CA samples, while in the NS there was only minimal p65 expression in the epithelial cell nuclei. ANOVA demonstrated that the p65 expression in the epithelial nuclei of VV and CA samples was significantly higher than in the NS (P<0.01). These findings indicate that p65 is transported to the nuclei due to NF-κB activation, and thus induces the expression of inflammatory factors, promoting cell proliferation and preventing apoptosis in the warts. Western blot analysis also demonstrated a significant p65 expression in the epithelial nuclei of CA and VV samples, whereas only a weak p65 expression in the NS was identified. NF-κB is sequestered and bound by the IkB family of inhibitor proteins in the cytoplasm in its inactive form. Various stimuli, including proinflammatory cytokines and viral replication are able to activate NF-κB by triggering the phosphorylation of IkB, and induce its subsequent molecular translocation to the nucleus. As expected, the immunohistochemistry and western blotting results proved that the level of p65 expression in the epithelial nuclei, which is indicative of NF-κB activation and inflammation responses, was increased and significantly higher in VV and CA than in the NS (P<0.01), suggesting that inflammation may be intensified in the HPV-infected epithelial cells, which may lead to the development of warts. The expression of p65 in the vascular endothelial cells in the VV and CA samples was significantly higher than in the NS (P<0.01). In addition, the p65 level in the vascular endothelial cells was positively correlated with the p65 level in the epithelial nuclei of the tissues (r=0.4355, P<0.01), indicating that NF-κB may activate the transcription and translation of genes to promote angiogenesis in the warts.

Furthermore, the expression of p65 in the associated inflammatory cell nuclei in the VV samples was higher than the other tissues (P<0.05). Together with the results of the HMGB1 inflammatory cell expression, it is deduced that the inflammation in VV may be mediated by inflammatory cells, and that the inflammation of CA may be induced by infected epithelial cells rather than inflammatory cells. Finally, correlation analysis demonstrated that the level of HMGB1 in epithelial intercellular spaces was positively correlated with the level of p65 in the epithelial nuclei (r=0.5199, P<0.01), further indicating that HMGB1 may be a mediator in inducing the local inflammation in the warts.

In conclusion, the present study demonstrated that inflammation was intensified in VV and CA samples. The HMGB1 and TLR4 signaling pathway-associated inflammation may have a central role in CA. Furthermore, HMGB1 may be one of mediators resulting in the progression of inflammation in VV, while the TLR4 signaling pathway does not appear to have a role in inducing inflammation in VV. Therefore, anti-HMGB1 therapy may be useful for the treatment of the warts caused by HPVs prior to their development into malignant lesions.

## Abbreviations

CA: condyloma acuminatum
HMGB1: high mobility group protein box 1
HPV: human papillomavirus
NF-κB: nuclear factor-κB
TLR4: toll like receptor 4
VV: verruca vulgaris
